# Characterization of Cytoskeletal Profilin Genes in Plasticity Elongation of Mesocotyl and Coleoptile of Maize Under Diverse Abiotic Stresses

**DOI:** 10.3390/ijms252111693

**Published:** 2024-10-30

**Authors:** Xiaoqiang Zhao, Siqi Sun, Zhenzhen Shi, Fuqiang He, Guoxiang Qi, Xin Li, Yining Niu

**Affiliations:** State Key Laboratory of Aridland Crop Science, College of Agronomy, Gansu Agricultural University, Lanzhou 730070, China; 15045240973@163.com (S.S.); shizz@gsau.edu.cn (Z.S.); hefq6125@163.com (F.H.); qigx1321@163.com (G.Q.); m18214360633@163.com (X.L.); niuyn@gsau.edu.cn (Y.N.)

**Keywords:** profilin, maize, mesocotyl, coleoptile, phylogenetic analysis, RNA-sequencing, quantitative real-time PCR

## Abstract

The plasticity elongation of mesocotyl (MES) and coleoptile (COL) largely determines the morphology of maize seedlings under abiotic stresses. The profilin (PRF) proteins play a pivotal role in cytoskeleton dynamics and plant development via regulating actin polymerization. However, little is known about whether and how the expression of the *ZmPRF* gene family regulates MES and COL elongation in maize under adverse abiotic stresses. Here, a total of eight *ZmPRF* gene members were identified in the maize genome. They were mainly located in the cytoplasm, chloroplast, and mitochondrion, and clearly divided into four classes, based on phylogenetic analysis. Segmental duplication was the main driver for the expansion of *ZmPRF* genes. Ka/Ks analysis indicated that most *ZmPRF* genes were intensely purified and selected. Promoter cis-element analysis suggested their potential roles in response to growth and development, stress adaption, hormone response, and light response. The protein–protein interaction network and two independent RNA-sequencing analyses revealed that eight *ZmPRF* genes and their thirty-seven interacting genes showed varied expression patterns in MES and COL of three maize genotypes under different sowing depths, 24-epibrassinolide application, and light spectral-quality treatments, of which *ZmPRF3.3* was a potential core conserved gene for breeding application. Moreover, the quantitative real-time PCR (qRT-PCR) verified that the relative expression levels of most *ZmPRF* genes in MES and COL under above treatments were significantly correlated with the plasticity elongation of MES and COL in maize. Therefore, these results perform a comprehensive overview of the *ZmPRF* family and will provide valuable information for the validation of the function of *ZmPRF* genes in maize development under diverse abiotic stress.

## 1. Introduction

Maize (*Zea mays*), is the most widely planted crop in the world, and is utilized globally as food, feed, and fuel, with a production of more than 1.14 billion tons in 2018 [1]. According to a forecast of the United Nations (https://www.fao.org/home/en/ (accessed on 8 September 2024)), the world’s population will exceed nine billion and global demand for maize will double, by 2050. In fact, maize generally grows in various harsh environment, such as drought, low/high temperature, and acid/saline–alkali soils, which seriously inhibits plant growth and reduces production [2,3,4,5,6]. Fortunately, maize has evolved a series of adaptive changes including morphology and physiology, and activated multiple stress-specific response genes and transcription factors (TFs) to response to stress regulations [7,8]. Considering their importance, more and more promising genes have been identified to understand how they respond to different stresses at transcriptional levels.

The actin cytoskeleton is the central player in the cytoskeleton, and displays crucial roles throughout the whole plant life cycle, such as cell expansion and division, vesicle trafficking, organelle movement, morphogenesis, and stress responses [9,10,11]. Interestingly, there are multiple proteins and factors taking part in these biological processes by regulating the formation of microfilaments and microtubules, actin nucleation and the homeostasis maintenance of globular (G-)actin [12,13]. In addition, the actin-binding proteins (ABPs) are an important class of proteins involved in the regulation of this dynamic rearrangement of action filaments [14], of which, profilin (PRF) is thought to be one of the major modulators [15].

The PRFs (Pfam accession number PF00235) have low molecular weight in both mono- and dicotyledonous, and they control action polymerization by ADP/ATP exchanges [16], and profilin/G-actin ratio, as well as ionic environment of cells [17]. Actin dynamics analysis revealed that PRFs bind to actin by forming a 1:1 complex with G-actin, suppress spontaneous actin nucleation, and inhibit monomer addition at filament pointed ends [18]. In vitro studies showed that PRF-actin complex could associate with the barbed ends of filaments, along with promoted actin polymerization, and that these changes were achieved by lowering the critical concentration and increasing nucleotide exchange on G-actin [19]. An early study [20] also suggested that PRFs had their molecular interactions mainly with polyphosphoinositides and proline-rich domain-containing proteins during cell wall development. In addition, PRFs participated in signal transduction, and may link transmembrane signaling to the control of the microfilament system [21]. In recent years, more than 400 *PRF* genes from different plant species have been found in the National Center for Biotechnology Information (NCBI) database, which were clearly divided into four classes [22]. Among them, *PRF1* in cotton (*Gossypium hirsutum*) regulated floral/apical meristem development and early-flowering phenotypes [16]. The inhibited *PRF-1* (*PFN-U*) *Arabidopsis thaliana* etiolated seedlings showed an overall dwarf phenotype, including short hypocotyls whose lengths were 20~25% that of the wild type at low temperatures [23]. Three *PRF* genes in rice (*Oryza sativa*) were involved in abiotic stresses (including NaCl, PEG6000, cold, light, and UV-B) and hormone response (i.e., indole-3-acetic acid (IAA), gibberellic acid 3 (GA_3_), abscisic acid (ABA), salicylic acid (SA), and brassinolide (BR)) processes [13]. A total of five *PRF* genes in maize have been identified so far, with distinct biochemical and live-cell properties [24,25], speculating that maize *PRF* genes may perform distinct functions. However, whether there are other *PRF* genes, and how these genes were expressed in different maize tissues under various environmental stresses, remain unclear.

The mesocotyl (MES) and coleoptile (COL) are important embryonic organs for sensing various stresses in maize, with their plasticity elongation thus influencing seedling emergence and morphology establishment [26]. Certainly, what we are interested in is whether *PRF* genes regulate plasticity elongation of MES and COL in maize under diverse stresses. Therefore, the aims of this study are to (1) identify *ZmPRF* gene members in maize and analyze their physicochemical properties, phylogenetic relationships, gene structure, conserved motifs, evolutionary selection patterns, duplication events, and protein interaction networks; (2) analyze gene expression patterns of *ZmPRFs* in both tissues under different treatments via RNA-sequencing (RNA-Seq); and (3) clarify the relationships between all *ZmPRF* expression and MES/COL plasticity elongation. These findings may provide valuable insights into studying the biology function of the *ZmPRF* family in maize and suggest their use for future breeding programs.

## 2. Results

### 2.1. Identification of PRF Family Members in Maize

**Figure 1:**
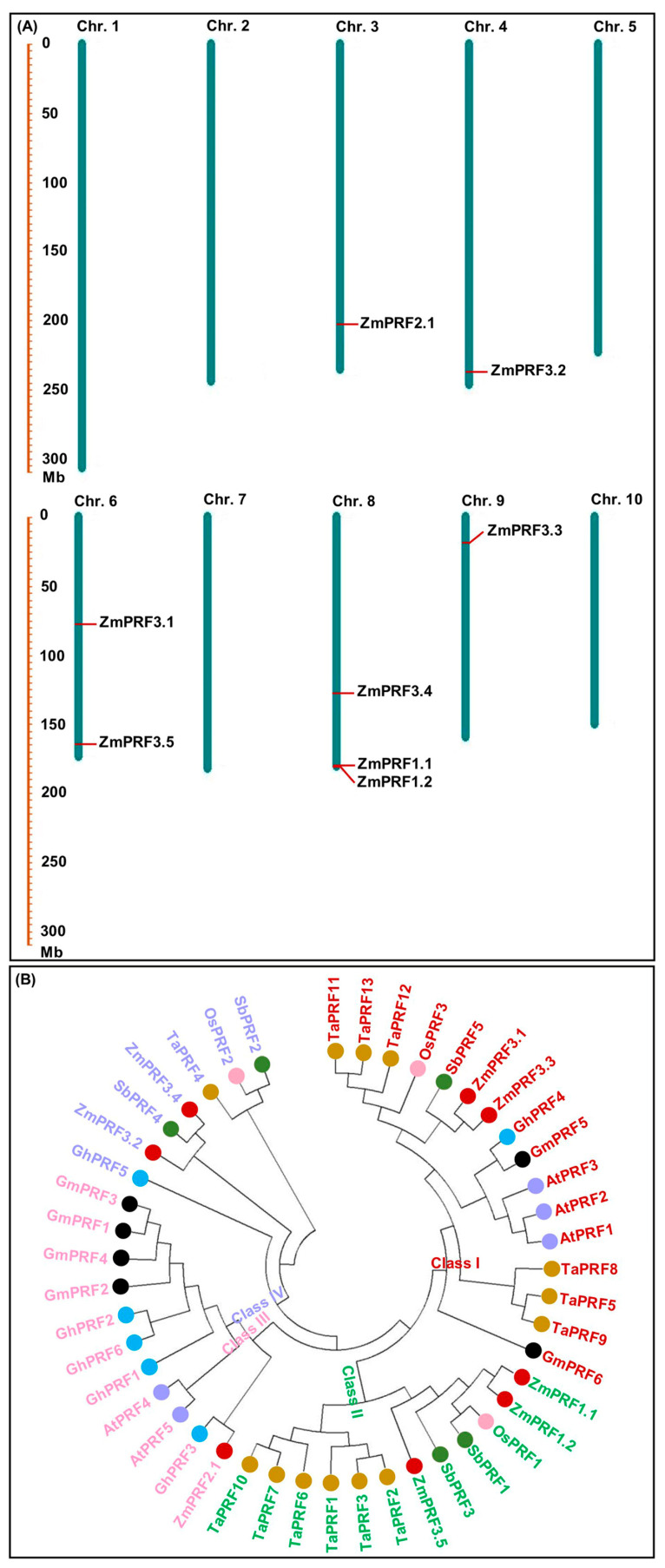
(**A**) Chromosomal locations of eight profilin (PRF) gene members detected in maize. (**B**) Phylogenic tree of PRF proteins from seven plant species, including *Arabidopsis thaliana* (At), *Glycine max* (Gm), *Triticum aestivum* (Ta), *Gossypium hirsutum* (Gh), *Sorghum bicolor* (Sb), *Zea mays* (Zm), and *Oryza sativa* (Os).

Using 31 PRF proteins sequences from *Arabidopsis thaliana* [27], *Oryza sativa* [13], and *Brassica juncea* [22], as a reference for the Hidden Markov Model (HMM) and BLASTP alignment, as a result, a total of eight *ZmPRF* members were identified in the maize genome (https://ftp.ensemblgenomes.ebi.ac.uk/pub/plants/release-60/fasta/zea_mays/dna/ (accessed on 16 April 2024)), and their names were assigned by the nomenclature of Khuman et al. [22] (Table 1). These *ZmPRF* members were then distributed on chromosome 3, 4, 6, 8, and 9 (Table 1; Figure 1A). Further analysis showed that these *ZmPRF* gene members encoded amino acids from 131 to 187, had protein molecular weights (MWs) varying from 14.11 to 19.34 kDa, and exhibited the theoretical isoelectric point (pI) values ranging from 4.59 to 6.29 (pI value < 7.0; Table 1), indicating that they may be acidic proteins. Meanwhile, except for *ZmPRF3.1*, the instability index of the other seven PRF proteins was less than 40 (Table 1), suggesting their stable nature. Moreover, the grand average of hydropathicity (GRAVY) values analysis indicated that only one protein, i.e., *ZmPRF3.5*, exhibited hydrophilic residues on its surface, whereas other members showed hydrophobic residues (Table 1). Additionally, all PRF proteins displayed diverse subcellular localizations, including cytoplasm, chloroplast, and mitochondrion (Table 1), showing their involvement in various cellular transport processes [22].

### 2.2. Phylogenetic Analysis of PRF Family

To further elucidate the evolutionary relationship of *ZmPRF* gene members in maize, a total of 46 *PRF* genes from seven plant species were selected (Table S1). There were significance differences in their amino acid number (ranging from 125 to 145, average 135), MWs (ranging from 13.62 to 15.76 kDa, average 14.58 kDa), and theoretical pI values (ranging from 4.86 to 5.59, average 5.16) among all species (Table S1). Using all PRF protein sequences, a neighbor-joining (NJ) phylogenetic tree was further constructed, and these *PRF* genes were then distinctly grouped into four classes (Figure 1B). Among them, Class I (consisting of 16 PRF protein members, including *ZmPRF3.1* and *ZmPRF3.3*), Class III (consisting of 11 PRF protein members, including *ZmPRF2.1*), and Class IV (consisting of 7 PRF protein members, including *ZmPRF3.2* and *ZmPRF3.4*) were commonly found in monocotyledonous and dicotyledonous species, while Class II consisted of 12 PRF protein members, including *ZmPRF1.1*, *ZmPRF1.2*, and *ZmPRF3.5* (Figure 1B). It is thus speculated that these PRF sequences may undergo significant structural transformations during the evolutionary split of monocotyledonous and dicotyledonous plants; at the same time, these transformations may have caused variations in the number of PRF sequences within each plant subfamily and species.

### 2.3. Gene Structures and Conserved Motifs of PRF Family

**Figure 2:**
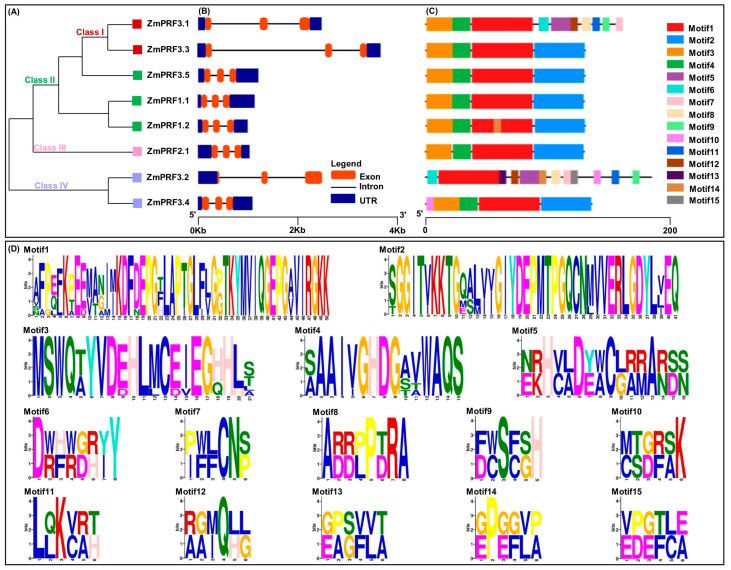
(**A**) Phylogenic tree of eight profilin (PRF) protein members in maize. (**B**) The exon–intron structure of these *PRF* gene members in maize. (**C**) Conserved motif distribution of all PRF protein members in maize. (**D**) The sequence logos of the 15 conserved motifs.

To gain insight into the architectures of maize *ZmPRF* gene members, exon–intron structure analysis was carried. The results showed that these genes contained three exons and two introns, and their untranslated region (UTR) lengths were clearly different (Figure 2B). Additional analysis of conserved motifs in maize *ZmPRF* members was conducted using the online tool Multiple Em for Motif Elicitation (MEME). The results revealed that a total of 15 conserved motifs were identified among all protein sequences, designated as Motif1–Motif15 (Figure 2C,D); of which, 4 (*ZmPRF1.1*, *ZmPRF2.1*, *ZmPRF3.3*, and *ZmPRF3.5*) to 12 (*ZmPRF3.2*) conserved motifs were detected in each protein (Figure 2C,D). Meanwhile, the same Motif1 was found in all PRF proteins, while some special conserved motifs, such as the Motif10 only existed in Class IV (*ZmPRF3.2* and *ZmPRF3.4*), and the Motif9 only arose in Class I (*ZmPRF3.1*) (Figure 2A,C,D). This suggested that these motifs may have evolved to perform specific functions unique to these classes, highlighting potential functional divergence among *ZmPRF* family members.

### 2.4. Cis-Element Analysis of PRF Family

**Figure 3:**
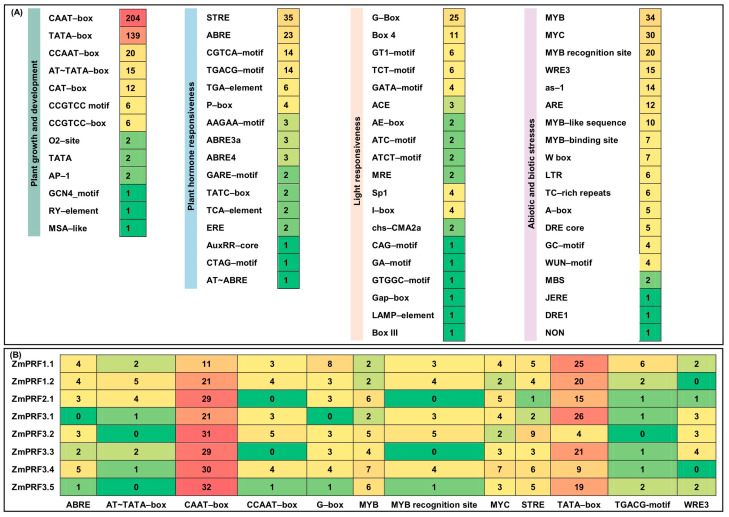
(**A**) Number statistics and element categories of predicted cis-elements in maize *profilin* (*PRF*) gene promoter regions. (**B**) Number statistics of the top 12 cis-elements in maize *PRF* gene promoter regions.

To predict possible functions and regulatory mechanisms of maize *ZmPRF* gene members, cis-elements within their 2 kb promoter-region sequence upstream of the start codon were analyzed. A total of 67 cis-elements were identified (Figure 3A). They were divided into 4 categories, including 13 growth-and-development-related elements, 19 stress-adaption elements, 16 hormone-response elements, and 19 light-response elements (Figure 3A). The findings suggested that these maize *PRF* genes likely interacted with multiple TFs. It is thus plausible to infer that maize *ZmPRF* genes may play a significant role in regulating multiple physiological processes, and that understanding their regulatory mechanisms could inform future studies focused on improving maize resilience and yield under varying environmental conditions. Moreover, we further obtained the top 12 important cis-elements, i.e., the growth-and-development-related elements of AT~TATA-box, CAAT-box, CCAAT-box, and TATA-box, the stress-adaption elements of the MYB, MYB-recognition site, MYC, and WRE3, the hormone-response elements of ABRE, STRE, and the TGACG-motif, as well as the light-response element of G-box (Figure 3B).

### 2.5. Collinearity Relationships of PRF Family

To investigate the evolution mechanisms of *PRF* gene members, we also analyzed intraspecific and interspecific collinearity analyses of all *PRF* genes. There was one collinearity relationship among eight *PRF* genes identified in the maize genome (Figure S1), suggesting that some maize *PRF* genes may have arisen from gene duplication events and that segmental duplication events are the main driver of maize *PRF* evolution. Moreover, only *ZmPRF2.1* was discovered to exhibit a collinear relationship with one *Sorghum bicolor SbPRF1* (the non-synonymous (Ka)/synonymous (Ks) value was 0.108); only *ZmPRF3.5* was found to have a collinear relationship with one *SbPRF3* (the Ka/Ks value was 0.120); only *ZmPRF3.4* was ascertained to show a collinear relationship with one *SbPRF2* (the Ka/Ks value was 0.061); *ZmPRF3.3* was detected to display a collinear relationship with one *Oryza sativa OsPRF3* (the Ka/Ks value was 0.962, and two *Triticum aestivum TaPRF11* (the Ka/Ks value was 0.996), and *TaPRK13* (the Ka/Ks value was 0.982) (Figure S2; Table S2), implying that these genes underwent evolutionary processes under purifying selection. Similarly, during the evolution of *Triticum aestivum* and *Sorghum bicolor*, *Triticum aestivum* and *Oryza sativa*, *Glycine max* and *Gossypium hirsutum*, *Arabidopsis thaliana* and *Brassica juncea*, 1, 3, 6, and 16 colinear gene pairs had Ka/Ks values less than 1, respectively (Figure S2; Table S2), indicating that natural selection favored the conservation of these genes throughout the evolutionary trajectory of both monocotyledonous and dicotyledonous species. However, *ZmPRF3.3* was found to have a collinear relationship with one *SbPRF5* (the Ka/Ks value was 1.017) and one *TaPRF12* (the Ka/Ks value was 1.006) (Figure S2; Table S2), suggesting that these genes were subjected to evolutionary processes under positive selection, which were thus retained during the evolution process and may contribute to their growth and development, as well as to adverse stress responses.

### 2.6. Protein–Protein Interaction (PPI) Networks Prediction

**Figure 4:**
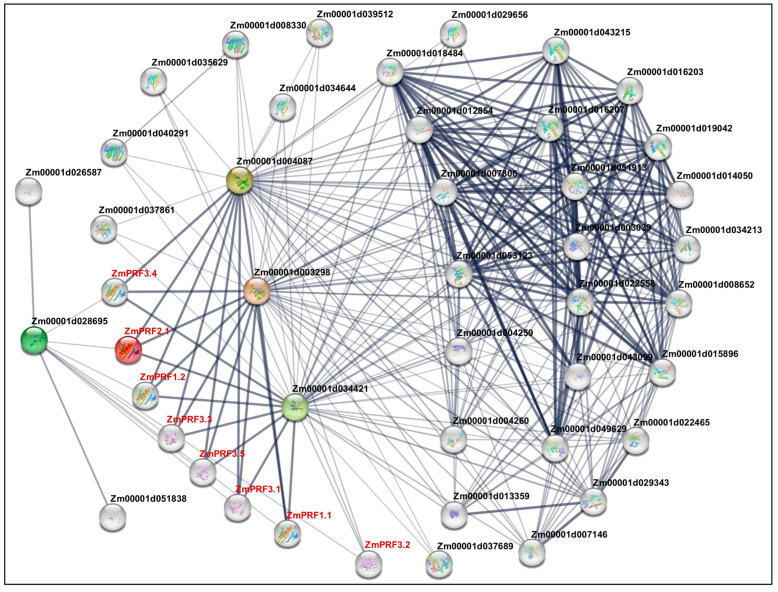
Protein–protein interaction network between profilin (PRF) proteins and other proteins in maize. Nodes represent proteins, and empty nodes are proteins with unknown 3D structures. Connections between nodes represent interactions between proteins, with edge thickness indicating the confidence level of the interaction. Query proteins are depicted in red-colored nodes.

The construction of PPI networks to link unknown functional proteins are beneficial for understanding the myriad biological activities and dynamic regulatory networks among biomolecules. In this study, we tried to explore potential PPI networks among PRF proteins in maize, including their direct and indirect associations using the STRING database (https://cn.string-db.org/ (accessed on 10 July 2024)). Based on the criteria of PPI enrichment *p*-value < 1 × 10^−16^ and an average local-clustering coefficient of 0.502, a total of thirty-seven functional proteins, including twenty-two actin (ACT), three F-actin, three actin-depolymerizing factors (ADFs), two dynein, three hexokinase, two zinc finger C-x8-C-x5-C-x3-H-type family proteins, one protein modifier of SNC1 11, and one unknown protein (*Zm00001d013359*) (Table S3), they showed direct and indirect association with eight PRF proteins (Figure 4). Subsequently, the Gene Ontology (GO) enrichment analysis of these 45 proteins was performed: the main categories were “actin filament depolymerization”, “negative regulation of actin filament polymerization”, “negative regulation of cytoskeleton organization”, “cell morphogenesis”, “negative regulation of organelle organization”, “negative regulation of cellular process”, “negative regulation of biological process”, and “response to stimulus” for the biological process, as well as the “cell cortex” and “cell periphery” for the cellular component (Figure S3). Thereby, these findings may reveal that they formed complex interaction networks to regulate cytoskeletal organization, actin filament dynamics, cell morphogenesis, and stress responses in maize.

### 2.7. PRF Gene Members Involved in Plasticity Elongation of Both Mesocotyl and Coleoptile in Maize Under Multiple Abiotic Stresses

The MES and COL are important tissues for early seedlings in maize to sense various abiotic stresses and signal molecules, determining the morphological formation of maize seedlings [26,28,29]. To reveal whether *ZmPRF* gene members regulated the plasticity elongation of both MES and COL in maize under different abiotic stresses, the following corresponding MES and COL were selected among different maize genotypes under diverse abiotic stresses to analyze the expression levels of eight *PRF* gene members by RNA-Seq and qRT-PCR analyses.

**Figure 5:**
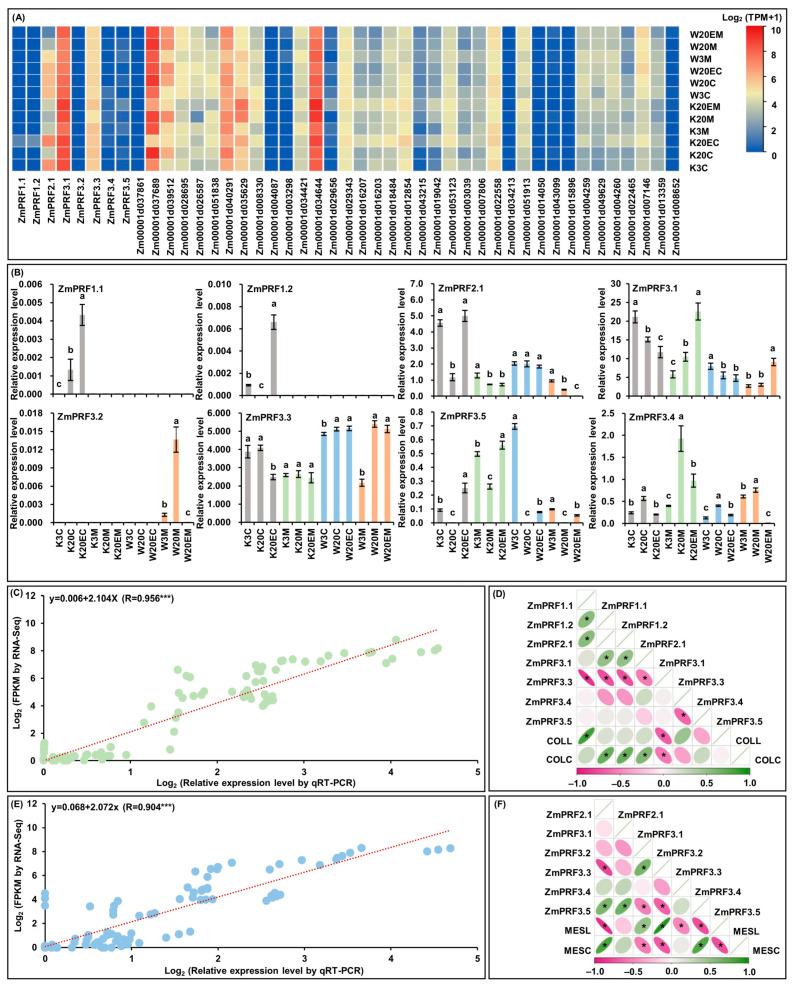
(**A**) Heatmaps of expression patterns from eight *profilin* (*PRF*) genes and their thirty-seven interacting genes in mesocotyl (MES) and coleoptile (COL) of W64A and K12 seedlings under three treatments by RNA-sequencing (RNA-Seq). W3M: MES of W64A seedlings at 3 cm sowing depth; W20M: MES of W64A seedlings at 20 cm deep-sowing stress; W20EM: MES of W64A seedlings were treated with 2.0 mg g^−1^ 24-epibrassinolide (EBR) application at 20 cm deep-sowing stress; W3C: COL of W64A seedlings at 3 cm sowing depth; W20C: COL of W64A seedlings at 20 cm deep-sowing stress; W20EC: COL of W64A seedlings were treated with 2.0 mg g^−1^ EBR application at 20 cm deep-sowing stress; K3M: MES of K12 seedlings at 3 cm sowing depth; K20M: MES of K12 seedlings at 20 cm deep-sowing stress; K20EM: MES of K12 seedlings were treated with 2.0 mg g^−1^ EBR application at 20 cm deep-sowing stress; K3C: COL of K12 seedlings at 3 cm sowing depth; K20C: COL of K12 seedlings at 20 cm deep-sowing stress; K20EC: COL of K12 seedlings were treated with 2.0 mg g^−1^ EBR application at 20 cm deep-sowing stress. TPM: transcripts per million. (**B**) The relative expression levels of eight *PRF* genes in MES and COL of W64A and K12 seedlings under three treatments by quantitative real-time PCR (qRT-PCR). Different lowercase letters in MES or COL in W64A/K12 seedlings under three treatments represent significant differences (*p* < 0.05) by analysis of variance (ANOVA). (**C**) Linear relationship between qRT-PCR and RNA-Seq for eight *PRF* genes in COL of two genotypes under three treatments. *** represents significant difference (*p* < 0.001) by ANOVA. (**D**) The Pearson correlation among eight *PRF* genes and two coleoptile phenotypes in two genotypes under three treatments. COLL: coleoptile length; COLC: coleoptile coarse. * represents significant correlations at *p* < 0.01 level. (**E**) Linear relationship between qRT-PCR and RNA-Seq for eight *PRF* genes in MES of two genotypes under three treatments. *** represents significant difference (*p* < 0.001) by ANOVA. (**F**) The Pearson correlation among eight *PRF* genes and two mesocotyl phenotypes in two genotypes under three treatments. MESL: mesocotyl length; MESC: mesocotyl coarse. * represents significant correlations at *p* < 0.01 level.

For RNA-Seq of MES and COL in W64A (deep-sowing tolerant genotype) and K12 (intolerant genotype) seedlings, that were cultured under two sowing-depth treatments and exogenous EBR stimulation for ten days [28,29]. Interestingly, the results showed that eight *PRF* genes members and their thirty-seven interacting genes had varied transcript-per-million (TPM) expression levels in both tissues of W64A and K12 seedlings under all treatments (Figure 5A; Table S4). At the same time, these *ZmPRF* gene members were then performed the qRT-PCR analysis in two tissues of both maize genotypes under three treatments (Figure 5B). The results showed that the qRT-PCR expression patterns were in agreement with eight *ZmPFR* genes in RNA-Seq dataset, and there was a good linear relationship between RNA-Seq expression levels and qRT-PCR expression patterns in MES (y = 0.068 + 2.072x; R = 0.904 ***)/COL (y = 0.006 + 2.104x; R = 0.956 ***) of both maize materials under all treatments (Figure 5C,E). According to the plasticity elongation phenotypes and *ZmPRF* qRT-PCR expression in MES and COL of W64A and K12 seedlings under all treatments, further Pearson correlation analysis showed that coleoptile length (COLL) was significantly correlated to the expression levels of *ZmPRF1.1* and *ZmPRF3.3*, and coleoptile coarse (COLC) was significantly correlated to the expression levels of *ZmPRF1.2*, *ZmPRF2.1*, *ZmPRF3.1*, and *ZmPRF3.3* (Figure 5D); similarly, mesocotyl length (MESL) was clearly correlated to the expression levels of *ZmPRF2.1*, *ZmPRF3.2*, *ZmPRF3.3*, *ZmPRF3.4*, and *ZmPRF3.5*, and mesocotyl coarse (MESC) was clearly correlated to the expression levels of *ZmPRF2.1*, *ZmPRF3.2*, *ZmPRF3.3*, and *ZmPRF3.5* (Figure 5F). These findings thus suggested that the activated or inhibited *ZmPRF* genes members could regulate the plasticity elongation of MES and COL in maize under deep-sowing stress and EBR stimulation.

**Figure 6:**
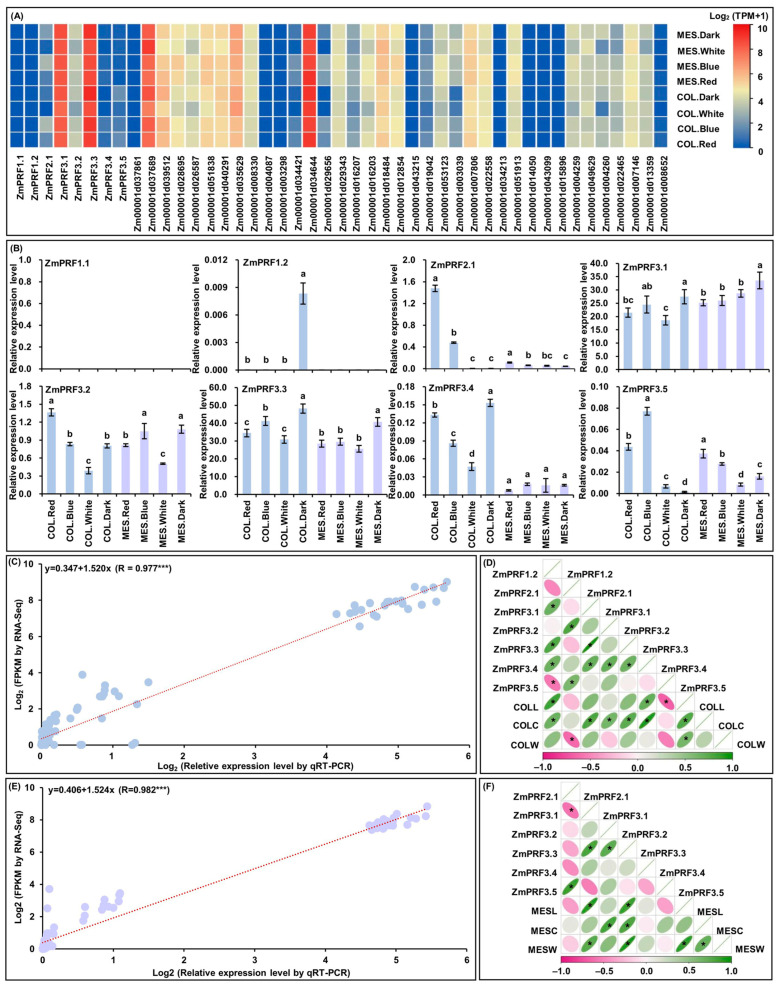
(**A**) Heatmaps of expression patterns from eight *profilin* (*PRF*) genes and their thirty-seven interacting genes in mesocotyl (MES) and coleoptile (COL) of Zheng58 seedlings under four treatments by RNA-sequencing (RNA-Seq). MES.Red: mesocotyl was cultured in red light; MES.Blue: mesocotyl was cultured in blue light; MES.White: mesocotyl was cultured in white light; MES.Dark: mesooctyl was cultured in darkness; COL.Red: coleoptile was cultured in red light; COL.Blue: coleoptile was cultured in blue light; COL.White: coleoptile was cultured in white light; COL.Dark: coleoptile was cultured in darkness. TPM: transcripts per million. (**B**) The relative expression levels of eight *PRF* genes in MES and COL of Zheng58 seedlings under four treatments by quantitative real-time PCR (qRT-PCR). Different lowercase letters in MES or COL in Zheng58 seedlings under four treatments represent significant differences (*p* < 0.05) by analysis of variance (ANOVA). (**C**) Linear relationship between qRT-PCR and RNA-Seq for eight *PRF* genes in COL of Zheng58 seedlings under four treatments. *** represents significant difference (*p* < 0.001) by ANOVA. (**D**) The Pearson correlation among eight *PRF* genes and three coleoptile phenotypes in Zheng58 seedlings under four treatments. COLL: coleoptile length; COLC: coleoptile coarse; COLW: coleoptile weight. * represents significant correlations at *p* < 0.01 level. (**E**) Linear relationship between qRT-PCR and RNA-Seq for eight *PRF* genes in MES of Zheng58 seedlings under four treatments. *** represents significant difference (*p* < 0.001) by ANOVA. (**F**) The Pearson correlation among eight *PRF* genes and three mesocotyl phenotypes in Zheng58 seedlings under four treatments. MESL: mesocotyl length; MESC: mesocotyl coarse, MESW: mesooctyl weight. * represents significant correlations at *p* < 0.01 level.

For RNA-Seq of MES and COL in Zheng58 seedlings, that were cultured four treatments for five days [26]. Intriguingly, the results showed that there were abundant TPM expression profiles of eight *ZmPRF* gene members and their thirty-seven interacting genes in both MES and COL of Zheng58 seedlings under all treatments (Figure 6A; Table S5). Subsequently, the qRT-PCR expression levels of these *ZmPRF* genes in MES and COL of Zheng58 under all treatments were further analyzed, and showing that the expression patterns from qRT-PCR and RNA-Seq analyses existed similar expression levels of up- or down-regulation (Figure 6B), and there was a good linear relationship between RNA-Seq dataset and qRT-PCR expression levels in MES (y = 0.406 + 1.524x; R = 0.982 ***)/COL (y = 0.347 + 1.520x; R = 0.977 ***) of Zheng58 seedlings under four light treatments (Figure 6C,E). Based on the plasticity elongation phenotypes and *ZmPRF* genes qRT-PCR expression in MES and COL of Zheng58 seedlings under four light treatments, their Pearson correlation analysis showed that COLL was significantly correlated to the expression levels of *ZmPRF1.2*, *ZmPRF3.4*, and *ZmPRF3.5*, COLC was significantly correlated to the expression levels of *ZmPRF1.2*, *ZmPRF3.1*, *ZmPRF3.2*, *ZmPRF3.3*, and *ZmPRF3.4*, and coleoptile weight (COLW) was significantly correlated to the expression level of *ZmPRF2.1* (Figure 6D); similarly, MESL was clearly correlated to the expression levels of *ZmPRF3.1* and *ZmPRF3.3*, MESC was clearly correlated to the expression levels of *ZmPRF3.2* and *ZmPRF3.3*, and mesocotyl weight (MESW) was clearly correlated to the expression levels of *ZmPRF3.1* and *ZmPRF3.3* (Figure 6F). These findings thus indicated that different light spectral-quality irradiation could induce the up-/down-regulation of *ZmPRF* genes members in both MES and COL of maize, to determine their plasticity elongation.

## 3. Discussion

It is reported that the *PRF* family is ancient, universal, and functionally diverged across kingdoms, and that it regulates various development processes of plant cells, especially cell wall maintenance through actin sequestering, nucleation and cytokinesis [30]. In long plant evolution, *PRF* sequences were identified in bryophytes and gymnosperms, which evolved approximately 400 and 300~325 million years ago, respectively, and these occurrences preceded the emergence of angiosperms, showcasing unexpectedly high functional homology [31]. In addition, their functional divergence in different species implied both subfunctionalization and neofunctionalization [32]. Based on these considerations, we identified eight *ZmPRF* gene members in maize genome and revealed their potential functions and evolution in the present study.

Our evolutionary analysis showed that forty-six PRF proteins from seven plant species, including both dicotyledons (*Arabidopsis thaliana*, *Brassica juncea*, *Gossypium hirsutum*, and *Glycine max*,) and monocotyledons (*Oryza sativa*, *Sorghum bicolor*, *Triticum aestivum*, and *Zea mays*), were clearly divided into four classes (Figure 1B), which was consistent with the phylogenetic tree analysis of PRF proteins in *Brassica* species [22]. These findings thus suggested that these *PRF* sequences from monocot species and dicot species have a common evolutionary origin. For example, *ZmPRF3.1* and *ZmPRF3.3* in Class I were more closely related to *Sorghum bicolor SbPRF5*, *ZmPRF1.1* and *ZmPRF1.2* in Class II were more closely related to *Oryza sativa OsPRF1*, *ZmPRF3.5* in Class II was more closely related to *SbPRF3*, *ZmPRF2.1* in Class III was more closely related to *Gossypium hirsutum GhPRF3*, and *ZmPRF3.4* and *ZmPRF3.2* in Class IV were more closely related to *SbPRF4* (Figure 1B). Except for *SbPRF2,* there existed two exon regions, and *SbPRF4* had five exon regions; other *PRF* genes members from seven species showed three exon regions (Table S1), and previous exon–intron analysis also reported that twenty-three *Brassica juncea PRFs* consisted of three exon regions [22]. Similarly, for these eight maize *PRF* genes, we identified three exon regions that were separated by intron regions of different sizes (Figure 2B). Moreover, *ZmPRF3.3* had the largest intron region among all maize *PRF* genes (Figure 2B). In addition, the UTR sequences, an important regulatory region for RNA transcription, RNA translation and RNA stability [32], also showed obvious differences among these maize *PRF* gene members (Figure 2B). This may be associated with the variation in expression patterns of the *PRF* genes in both maize tissues under different abiotic stresses and hormone treatments.

In *Brassica juncea,* except for *BjPRF3-4* that was a hydrophobic protein, other 22 *BjPRF* members were hydrophilic proteins [22]. Unlike dicot *Brassica juncea*, in monocot maize, nearly 90% (seven) PRF proteins showed hydrophobic proteins (Table 1). It is thus speculated that the hydrophobicity of PRF proteins in monocot species may drive the folding of proteins’ complex structures, forming their stable structures [33]. As is well known, the primary forces driving the emergence of new family members and novel functions in plant evolution are undergoing segmental duplication and tandem duplication [34]. In this study, only one pair of segmentally duplicated genes was identified in maize *PR*F gene members (Figure S1). This implied that the evolution of maize *PRF* genes was dominated by segmental duplication, which may play a pivotal role in the expansion of the *PRF* gene family in maize. Certainly, our genomic collinearity analysis of the *PRF* family from seven species identified numerous collinear gene pairs, the presence of collinear relationships in *Zea mays* and *Sorghum bicolor* with three pairs, *Zea mays* and *Oryza sativa* with one pair, *Zea mays* and *Triticum aestivum* with two pairs, *Triticum aestivum* and *Sorghum bicolor* with one pair, *Triticum aestivum* and *Oryza sativa* with three pairs, and *Glycine max* and *Gossypium hirsutum* with six pairs, as well as *Arabidopsis thaliana* and *Brassica juncea* with sixteen pairs (Figure S2; Table S2). Thereby, these data suggest that the *PRF* family are highly conserved. Additionally, subcellular localization prediction can locate a certain protein or expression product at a cell-specific location, providing a research direction for elucidating gene mechanisms [35]. Like twenty-three PRF proteins from *Brassica juncea*, eight maize PRF proteins were mainly located in the chloroplast, cytoplasm, and mitochondrion (Table 1), indicating that *PRF* genes act on these organelles and perform different biological functions in various plant species. Previously, Park et al. [36] also reported that when plants respond to the innate immunity response, chloroplasts generated tubular structures to facilitate chloroplast movement towards the nuclei, and the microtubules and actin filaments provided direction and driving force during these changes. Notably, to further verify the results of subcellular prediction, it is necessary to analyze the instantaneous expression of *ZmPRFs* in tobacco leaves using Agrobacterium-mediated methods and observe the fluorescence signals by laser confocal microscopy, in the future.

Initially, identifying their role in actin sequestration/binding, which thus actively contributes to the dynamics of actin polymerization [22], increasing evidence has revealed diverse functions of PRF proteins across different plant species, such as increased *PRF* transcription correlated with a proportional improvement in *F-actin* levels by a RNA-Seq analysis of 10-day post-anthesis fiber tissues across different cotton cultivars, resulting in regulating cotton-fiber elongation [37]. Overexpression of *AtPRF3* resulted in significant decreases in root length and hypocotyl length, and in delayed seed germination in *Arabidopsis thaliana* [38]. Using *pronp1* (a *PRF* gene) transgenic *Nicotiana tabacum* plants, revealed a prominent expression of *pronp1* in mature pollen and elongating pollen tubes, and significant activity in the root hairs of developing seedlings [39]. Domestication-driven *GhPRF1* transduced the early-flowering phenotype in *Nicotiana tabacum* by spatial alteration of apical/floral-meristem-related gene expression [16]. *Triticum aestivum PRFs* had aberrant distribution in root-tip cells of seedlings exposed to enhanced UV-B radiation, influencing the cell-elongating axis during the telophase [40]. The specific members of *PRFs* and *ADFs* might participate in regulating the response of wheat to low-temperature stress [41]. Similarly to the above previous studies, our promoter cis-elements of maize *PRF* genes confirmed that these genes may be involved in maize growth and development, stress adaption, light response, and hormone stimulation (Figure 3). Moreover, it is possible to form a complex interaction network among multiple PRF, ACT, F-actin, ADF, denein, and hexokinase proteins, to control various functions in maize (Figure 4).

**Figure 7:**
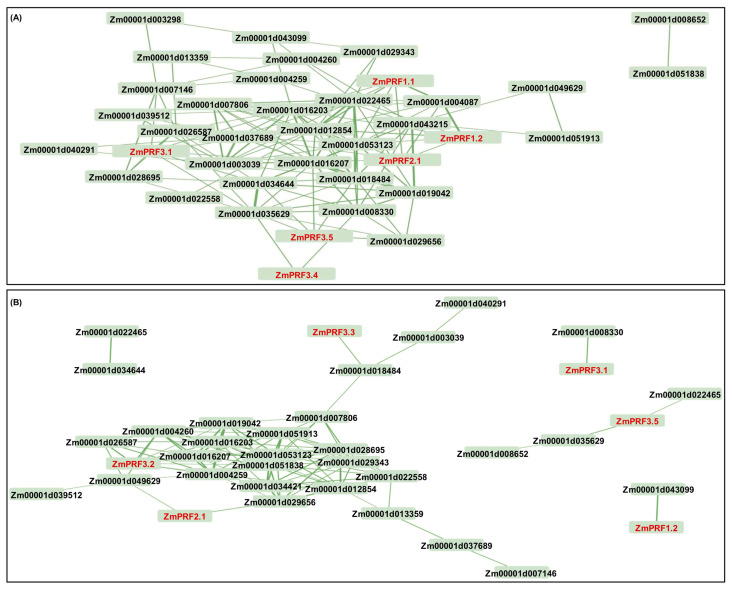
(**A**) Based on the fragments-per-kilobase-per-million mapped (FPKM) values of RNA-sequencing (RNA-Seq) for mesocotyl (MES) and coleoptile (COL) in W64A and K12 seedlings under three treatments, including 3 cm sowing depth, 20 cm sowing depth, 2.0 mg g^−1^ 24-epibrassinolide (EBR) was applied at 20 cm sowing depth, the interaction network mapping was constructed between *profilin* (*PRF*) and other corresponding genes in maize. (**B**) Based on the FPKM values of RNA-Seq for MES and COL in Zheng58 seedlings under four treatments, including red light, blue light, white light, and darkness, the interaction networks mapping was constructed between *PRF* and other corresponding genes in maize.

Further, we found that these *ZmPRFs* showed positive/negative expression patterns, and they even interacted with multiple proteins to regulate the plasticity elongation of MES and COL in maize under diverse deep-sowing stress, exogenous EBR application, and light spectral-quality irradiation (Figure 5, Figure 6 and Figure 7). For example, the *ZmPRF1.1* expression level was significantly up-regulated in COL of the K12 genotype under 20 cm deep-sowing stress and 2.0 mg g^−1^ exogenous EBR stimulation, to positively regulate the length of the coleoptile in K12, implying that this gene shows tissue-expression specificity. *ZmPRF3.3* showed varied expression patterns in MES and COL of three genotypes (W64A, K12, and Zheng58) under diverse deep-sowing stress, exogenous EBR application, and light spectral-quality irradiation; which was interacted with *Zm00001d018484* (ACT), subsequently, their cooperation regulated the plasticity elongation of MES and COL in maize exposed to different light spectral conditions (Figure 7), including the remodeling of MES and COL phenotypes, as a potential core-conserved gene in the future. In addition, *ZmPRF3.4* transcription showed significantly positive correlation with the length of the mesocotyl in maize under deep-sowing stress and exogenous EBR application; in contrast, it showed a clearly negative correlation with the length and coarse of coleoptile in maize under various light spectral-quality conditions. However, the observed diverse functions of *ZmPRFs* highlight the necessity for further exploration to unravel the intricate molecular mechanisms governing the involvement of *ZmPRFs* in other growth and development processes, while it is worth noting that we need to further verify their interaction relationships among *ZmPRFs* and other genes with yeast two-hybrid tests, in the future.

## 4. Materials and Methods

### 4.1. Genome-Wide Identification of PRF Gene Members

Five *AtPRF* genes were obtained from the *Arabidopsis thaliana* genome database (https://www.arabidopsis.org/ (accessed on 16 April 2024)), three *OsPRF* genes were downloaded from the *Oryza sativa* genome database (http://rice.plantbiology.msu.edu/ (accessed on 16 April 2024)), and twenty-three *BjPRF* genes were collected from the *Brassicaceae* genome database (http://brassicadb.cn/#/ (accessed on 16 April 2024)). We then used the HMM profile of the specific “PF00235” domain in the Pfam database (http://pfam.xfam.org/ (accessed on 16 April 2024)) to identify eight *ZmPRF* genes from the maize genome (http://ftp.ensemblgenomes.org/pub/plants/release-50/fasta/zea_mays/dna/ (accessed on 16 April 2024)) with the HMMER 3.0 software (http://hmmer.org/download.html (accessed on 20 April 2024)). Next, BLASTP comparisons were performed as a query, with the E-value < 1 × 10^−5^ [42]. After removing all redundant sequences, these *ZmPRF* genes were named using the nomenclature of Khuman et al. [22].

### 4.2. Sequence Analysis, Structural Characterization, Subcellular Localization Prediction, and Phylogenetic Tree of PRF Genes

The CDS, gDNA, and protein sequences of all *PRF* gene members from seven species were downloaded from the NCBI public database (https://www.ncbi.nlm.nih.gov/ (accessed on 11 May 2024)), respectively. The amino acid numbers, MW, theoretical pI value, instability index, aliphatic index, and GRAVY of these PRF proteins were further determined using the ExPASy (https://web.expasy.org/protparam/ (accessed on 13 May 2024)). The MG2C v.2.1 (http://mg2c.iask.in/mg2c_v2.1/ (accessed on 14 May 2024)) [43] was utilized to generate a physical map depicting the localization of *PRF* genes on maize chromosomes. The exon–intron structure of all maize *ZmPRFs* were displayed by the Gene Structure Display Server (GSDS2.0; http://gsds.cbi.pku.edu.cn/ (accessed on 15 May 2024)). The conserved motifs of corresponding PRF proteins were predicted via the MEME suite program v.5.0.5 (http://meme-suite.org/ (accessed on 4 June 2024)), with the maximum number of motifs set to 15 [44]. The subcellular localization prediction of these PRF proteins was also performed, using the Plant-mPLOC program (http://www.csbio.sjtu.edu.cn/bioinf/plant-multi/ (accessed on 19 June 2024)). The NJ phylogenetic tree of all PRF proteins was further conducted using the molecular evolutionary genetics analysis software (MEGA 6.0; https://www.megasoftware.net/ (accessed on 29 June 2024)) [45].

### 4.3. Cis-Element Analysis of PRF Genes

The PlantCARE online tool (http://bioinformatics.psb.ugent.be/webtools/plantcare/html/ (accessed on 3 July 2024)) [46] was used to analyze the initiation codon (ATG) 2000 bp upstream sequence of the promoter sequence from eight maize *PRF* gene members.

### 4.4. Duplication Events and Collinearity Analysis of PRF Genes

The MCscanX v.1.5.1 (https://help.rc.ufl.edu/doc/MCScanX (accessed on 2 July 2024)) [47] was used to detect maize *PRF* duplication and collinearity analysis, and Circos v.069 (https://circos.ca/ (accessed on 5 July 2024)) [48] was applied to provide a visual representation of the synteny blocks between *PRF* genes from seven species, including *Arabidopsis thaliana*, *Brassica juncea*, *Gossypium hirsutum*, *Glycine max*, *Oryza sativa*, *Sorghum bicolor*, *Triticum aestivum*, and *Zea amys*. Following that, the KaKs_Calculator 2.0 (https://sourceforge.net/projects/kakscalculator2/ (accessed on 11 July 2024)) [49] was employed for Ka and Ks substitution rates for each duplicated pair of *PRF* genes.

### 4.5. Protein–Protein Interaction (PPI) Network Prediction and Their GO Annotation

The STRING v.12.0 (https://cn.string-db.org/ (accessed on 24 July 2024)) was used to construct a PPI network among PRF proteins and other proteins in maize [50]. The STRING database can systematically collect and integrate PPI in both physical interactions and functional associations, and provides tools for GO enrichment analysis and Kyoto Encyclopedia of Genes and Genomes (KEGG) pathway analysis [50]. In this study, k-means clustering from STRING was utilized to cluster genes based on the evidence score, with a PPI enrichment *p*-value < 1 × 10^−16^ and an average local-clustering coefficient of 0.502.

### 4.6. Expression Patterns of PRF Genes and Their Interacting Genes Under Diverse Abiotic Stresses

For our previous studies [28,29], briefly, the 100 mL of 0.0 (ddH_2_O) and 2.0 mg g^−1^ EBR solution was mixed with 100 g dry vermiculite to prepare two cultivation substrates, respectively, which were then put into PVC tubes (50 cm height, 17 cm diameter); next, 30 soaked seeds (with 2.0 mg g^−1^ EBR solution or ddH_2_O for 24 h in darkness, respectively) of W64A and K12 were sown in corresponding PVC tubes that contained 2.0 mg g^−1^ EBR solution and ddH_2_O cultivation substrates at 3 cm and 20 cm sowing depths, respectively. In total, there were three treatments, i.e., 3 cm sowing depth + 0 mg g^−1^ EBR solution, 20 cm sowing depth + 0 mg g^−1^ EBR solution, and 20 cm sowing depth + 2.0 mg g^−1^ EBR solution. These were then cultured in a greenhouse (22 ± 0.5 °C, with 12/12 h light/dark cycle, and 60% relative humidity) for 10 days, and 40 mL corresponding EBR solution was added at 2-day intervals. Then MES and COL of W64A and K12 seedlings under three treatments, with three replicates, 36 samples in total, were used to perform RNA-Seq and measure their phenotypes, including MESL, COLL, MESC, and COLC, respectively [28,29].

For our previous study [26], briefly, the sterilized seeds of Zheng58 (i.e., the female parent of Zhengdan958 cultivar, China [51]) were soaked for 24 h in darkness, and were then pre-cultured in germinating boxes for five days at 22 ± 0.5 °C in darkness. Next, these etiolated seedlings were continuously cultured in plant chambers and illuminated with lamps consisting of three light-emitting diode (LED) bars specifically designed to provide a custom spectrum, i.e., red light: peak wavelength 660 nm, photosynthetic photon flux density (PFD) 22 µM m^−2^ s^−1^, 12 h photoperiod, blue light: peak wavelength 450 nm, PFD 13 µM m^−2^ s^−1^, 12 h photoperiod, and white light: PFD 17 µM m^−2^ s^−1^, 12 h photoperiod, in each chamber, respectively. Control etiolated seedlings were still cultured in darkness. The 20 mL Hoagland solution was added to each box at 2-day intervals, and to the other culture environment with 22 ± 0.5 °C, and 70% relative humidity was maintained. Then, MES and COL of Zheng58 seedlings under four treatments, with three biological replicates, a total of twenty-four samples, were used to perform RNA-Seq and measure their phenotypes, including MESL, COLL, MESC, COLC, MESW, and COLW, respectively [26].

After filtering, all clean reads were obtained and aligned to the *Zea mays* B73_v4 reference genome (ftp://ftp.ensemblgenomes.org/pub/plants/release-6/fasta/zea_mays/dna/ (accessed on 18 December 2023)) using HISAT 2.2.1 (http://ccb.jhu.edu/software/hisat2 (accessed on 18 December 2023)). The corresponding data were then analyzed using HTSeq v.0.9.0 (http://htseq.readthedocs.io/en/release_0.9.1/ (accessed on 18 December 2023)) based on the read count data obtained from expression profiling and calculated fragments per-kilobase-per-million mapped (FPKM) [52]. Meanwhile, their transcripts-per-million (TPM) values of corresponding genes based on the normalized scale method were calculated [53]. The gene-expression profiling was visualized using TBtools v.2.030 software (https://github.com/CJ-Chen/TBtools-II/releases (accessed on 3 August 2024)), according to the above predicted results of the PPI networks, using the FPKM expression patterns of these PPI genes from our independent RNA-Seq analyses to verify their interaction networks, using Cytoscape v.3.7.1 (https://cytoscape.org/ (accessed on 25 August 2024)).

### 4.7. qRT-PCR Analysis of PRF Genes

The same total RNA as our previous RNA-Seq [26,28,29] was reverse-transcribed to produce first-strand cDNA using the PrimeScript^TM^ first-strand cDNA synthesis Kit (TaKaRa, Japan). The special primers of eight *ZmPRF* genes were designed using Primer3web v.4.1.0 (https://primer3.ut.ee/ (accessed on 16 August 2024)) (Table S6). The LightCycler480II fluorescent quantitative PCR instrument (Roche, Munich, Germany) was used for *ZmPRF* qRT-PCR analysis. *Actin-1* (*Zm00001d010159*) was used as the internal reference gene [26]. There were three replicates for gene relative-expression analysis, and the relative gene-expression level was calculated by the 2^−ΔΔCT^ method [26].

### 4.8. Statistical Analyses

The analysis of variance (ANOVA) of qRT-PCR expression levels of eight *ZmPRF* genes was performed using IBM-SPSS Statistics v.19.0. The linear-relationship analysis between qRT-PCR expression levels and RNA-Seq FPKM expression patterns among all *ZmPRF* genes was performed by IBM-SPSS Statistics v.19.0. Further, all phenotype observations of MES and COL [26,28,29] and the corresponding qRT-PCR expression levels of these *ZmPRF* genes were used to analyze their Pearson correlation relationships and to visualize them via the online Genescloud tool (https://www.genescloud.cn (accessed on 18 August 2024)).

## 5. Conclusions

This study provided the comprehensive identification and analysis of *ZmPRF* gene members in maize. A total of eight identified *ZmPRF* gene members were unevenly distributed on five chromosomes. The analyses of gene structure, conserved motifs, and the phylogenetic tree showed that these *ZmPRFs* sequences were conserved, structurally similar, and divided into four categories. Notably, segmental duplication was found to be the main reason for family expansion during the evolution of maize *ZmPRFs*. Further Ka/Ks analysis revealed that most *ZmPRF* genes were intensely purified and selected. PPI prediction found that thirty-seven corresponding proteins were interacted with eight PRF proteins in maize, which were involved in actin cytoskeleton regulation, cell morphogenesis, and stress responses in maize. Multiple growth-and-development elements, stress-adaption elements, hormone-response elements, and light-response elements were identified in the promoter region. Moreover, by combining multiple analyses including RNA-Seq, qRT-PCR, Pearson correlation, and interaction network mapping, we confirmed that most *ZmPRFs* and their multiple interacting genes formed a complex interaction network to regulate MES/COL plasticity elongation in maize under various sowing depths, EBR application, and light spectral-quality treatments. These results thus lay the foundation for further studies on the functions of the *ZmPRF* family in plasticity elongation of MES and COL and abiotic-stress responses, which may have potential for application to maize breeding with stress resistance.

## Figures and Tables

**Table 1:** Genomic information and protein characteristics of eight profilin (PRF) gene family members in maize.

Nomenclature	Gene_ID	Chr.	Position (bp)	Amino Acid (No.)	MW (Da)	pI	Instability Index	Aliphatic Index	GRAVY	Subcellular Localization Predictions
*ZmPRF2.1*	*Zm00001d043523*	3	202,712,631–202,713,672	132	14,336.5	4.94	26.10	81.21	0.057	Cytoplasm
*ZmPRF3.2*	*Zm00001d053631*	4	237,406,007–237,408,981	187	19,343.9	4.81	35.32	73.05	0.147	Chloroplast
*ZmPRF3.1*	*Zm00001d036213*	6	77,268,316–77,270,830	161	18,356.8	6.29	45.60	76.89	0.376	Cytoplasm
*ZmPRF3.5*	*Zm00001d038783*	6	164,327,814–164,329,029	131	14,121.2	4.94	32.16	82.67	–0.102	Cytoplasm
*ZmPRF3.4*	*Zm00001d010797*	8	128,115,856–128,116,962	137	14,811.0	5.09	35.45	74.82	0.166	Mitochondrion
*ZmPRF1.1*	*Zm00001d012772*	8	180,571,569–180,572,723	131	14,265.4	4.91	29.63	75.11	0.115	Cytoplasm
*ZmPRF1.2*	*Zm00001d012773*	8	180,621,908–180,623,041	132	14,365.5	4.91	33.30	84.17	0.073	Cytoplasm
*ZmPRF3.3*	*Zm00001d045323*	9	18,935,792–18,946,869	131	14,114.2	4.59	34.03	87.02	0.044	Cytoplasm

Chr.: chromosome; MW: molecular weight; pI: theoretical isoelectric point; GRAVY: grand average of hydropathicity.

## Data Availability

Data are contained within the article and Supplementary Material.

## Supplementary Materials

The following supporting information can be downloaded at: https://www.mdpi.com/article/10.3390/ijms252111693/s1.
