# Supplementary material for: Characterization of Cytoskeletal Profilin Genes in Plasticity Elongation of Mesocotyl and Coleoptile of Maize Under Diverse Abiotic Stresses
Source: Int J Mol Sci. 2024 Oct 30;25(21):11693. doi: 10.3390/ijms252111693 (PMC11546416; doi:10.3390/ijms252111693)
Supplement: Supplementary file 1 [file ijms-25-11693-s001.zip › Supplementary Figures.pdf]

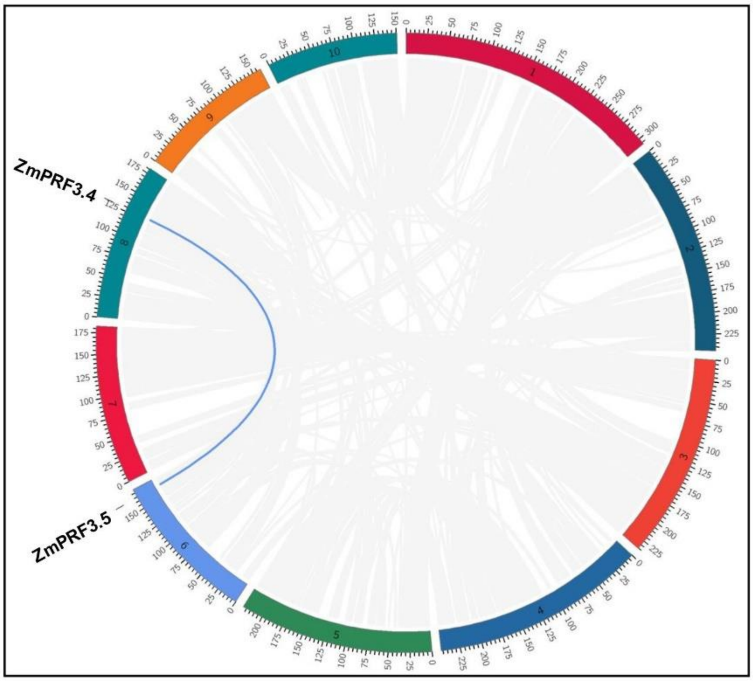

Figure S1. The intraspecific collinearity relationships of maize eight *profilin* (*PRF*) genes. Segmental duplicated gene pairs were represented by blue lines between chromosomes.

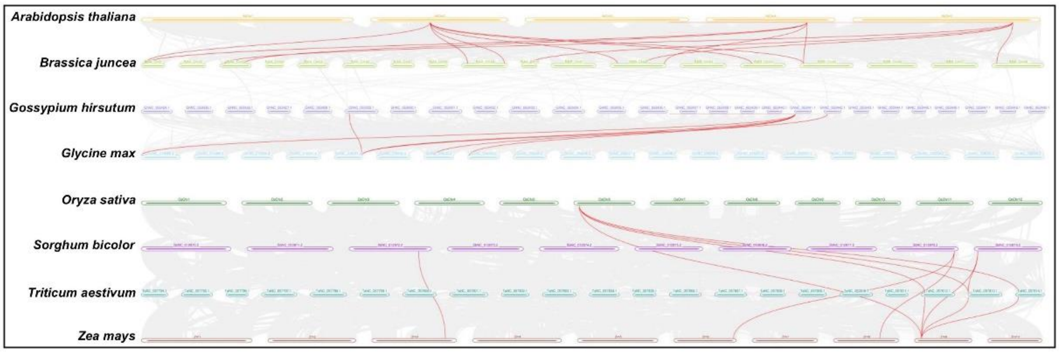

Figure S2. The interspecific collinearity relationships of *profilin* (*PRF*) genes in *Arabidopsis thaliana*, *Brassica juncea*, *Gossypium hirsutum*, *Glycine max*, *Oryza sativa*, *Sorghum bicolor*, *Triticum aestivum*, and *Zea amys*. The collinear blocks were shown by red lines.

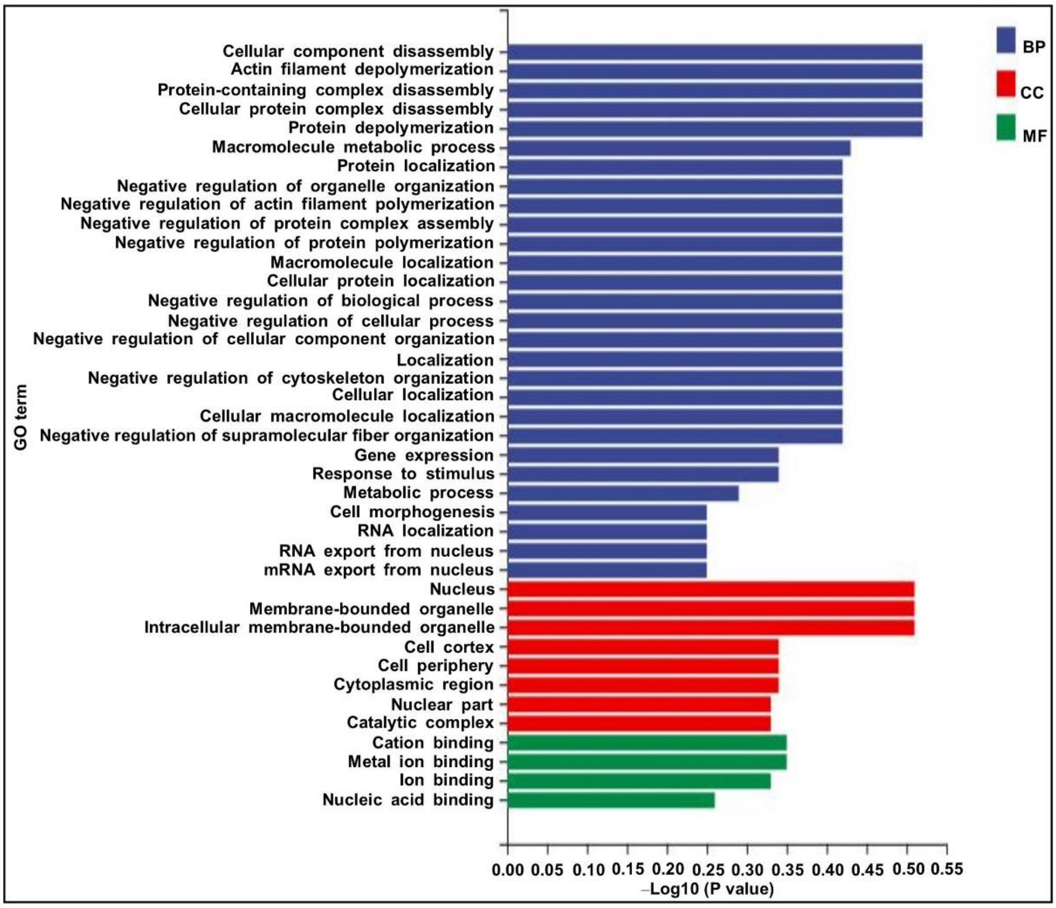

Figure S3. The 40 top gene ontology (GO) enrichment analysis of 45 proteins, including biological process (BP), cellular component (CC), and molecular function (MF).
